# The 100 most cited articles in artificial intelligence related to orthopedics

**DOI:** 10.3389/fsurg.2024.1370335

**Published:** 2024-04-17

**Authors:** Necmettin Turgut, Salih Beyaz

**Affiliations:** Department of Orthopedics and Traumatology, Adana Turgut Noyan Research and Training Centre, Başkent University, Adana, Türkiye

**Keywords:** machine learning, deep learning, decision support, artificial intelligence, classification

## Abstract

**Background:**

This bibliometric study aimed to identify and analyze the top 100 articles related to artificial intelligence in the field of orthopedics.

**Methods:**

The articles were assessed based on their number of citations, publication years, countries, journals, authors, affiliations, and funding agencies. Additionally, they were analyzed in terms of their themes and objectives. Keyword co-occurrence, co-citation of authors, and co-citation of references analyses were conducted using VOSviewer (version 1.6.19).

**Results:**

The number of citations of these articles ranged from 32 to 272, with six papers having more than 200 citations The years of 2019 (*n*: 37) and 2020 (*n*: 19) together constituted 56% of the list. The USA was the leading contributor country to this field (*n*: 61). The most frequently used keywords were “machine learning” (*n*: 26), “classification” (*n*: 18), “deep learning” (*n*: 16), “artificial intelligence” (*n*: 14), respectively. The most common themes were decision support (*n*: 25), fracture detection (*n*: 24), and osteoarthrtitis staging (*n*: 21). The majority of the studies were diagnostic in nature (*n*: 85), with only two articles focused on treatment.

**Conclusions:**

This study provides valuable insights and presents the historical perspective of scientific development on artificial intelligence in the field of orthopedics. The literature in this field is expanding rapidly. Currently, research is generally done for diagnostic purposes and predominantly focused on decision support systems, fracture detection, and osteoarthritis classification.

## Introduction

The concept of artificial intelligence (AI) was initially proposed by McCarthy, a cognitive and computer scientist, in 1956 (1). Since then, as an innovative technology, the application of artificial intelligence in various fields, including medicine and orthopedics, has garnered significant attention. In the near future, the impact of artificial intelligence applications could lead to a significant transformation in the practice of orthopedics (2). Artificial intelligence in orthopedics can yield benefits in areas such as preoperative diagnosis, patient selection, outcome and complication prediction, data tracking optimization, and the intraoperative surgical performance (2, 3).

Robot-assisted surgeries, navigated or computer-assisted surgeries in orthopedics are being applied in many places and their areas of use are expanding (4, 5). In addition to this, semi-automated or fully automated preoperative planning and templating systems are also available (6). It is difficult not to foresee an increase in AI usage in orthopedics over time, similar to its expansion in other fields. The importance of data has increased during this period, and the data obtained preoperatively, intraoperatively, and postoperatively has grown. Evaluating such a large amount of data has become difficult for humans, and the presence of AI-based systems is necessary in a much shorter timeframe for processing this data which is called big data. These systems save time for orthopedists and increase the accuracy rate in diagnosis and treatment, thereby enhancing the surgeon's chances of success. However, in order for orthopedists to be able to delegate this to AI-enhanced systems, they must first have basic knowledge on this subject. In this rapidly evolving field, mastering knowledge has also become more challenging. Therefore, gaining access to the most impactful and essential articles in this field has become crucial.

The increasing number of publications in the field of artificial intelligence-related orthopedics has made it challenging to grasp the subject and the literature comprehensively. Consequently, students, orthopedic residents, expert physicians, and engineers engaged in this field feel the need to delve into the most significant works primarily. Citation analysis is one of the most critical parameters illustrating a study's significance and influence within the academic community. Citation analyses are brought to light through bibliometric studies. Bibliometric analysis involves the computer-aided investigation of literature published in a specific topic or field using an appropriate methodology. Through these analyses, aspects such as the impact, content, citations received, authors, and finer details of articles are meticulously examined. The number of citations an article receives is a crucial measure of its influence.

Similar bibliometric studies have been conducted in orthopedics and other medical fields (7–9). However, as far as we know, this is the first bibliometric analysis focusing on the highest-impact articles within the field of orthopedics, those related to artificial intelligence. In this study, our objective was to identify and conduct a bibliometric analysis of the top 100 articles with the highest number of citations in the field of orthopedics, specifically those related to artificial intelligence. Our hypothesis was that due to the accelerated growth of studies related to artificial intelligence, newer articles might find a more prominent place in the list.

## Materials and methods

**Figure 1:**
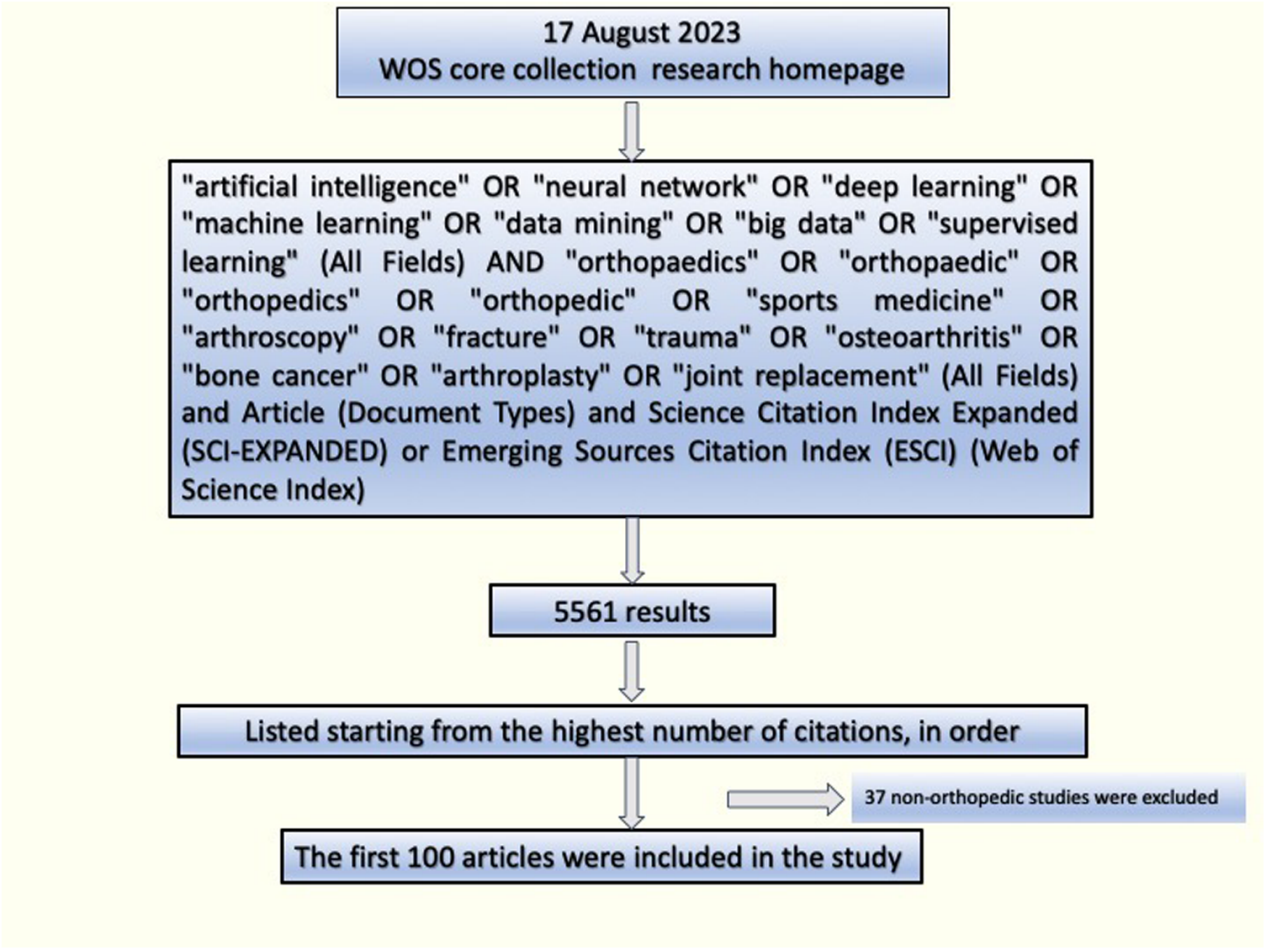
Search diagram.

Ethical committee approval was not sought for this study, as it was a bibliometric study and involving no human subjects. The data were collected using the Thomson ISI Web of Science Core Collection (WOSCC). The reason for using Web of Science in this article to obtain the data is its greater consistency and reliability than other databases, making it the primary choice for similar bibliometric studies in the literature (9, 10). All relevant articles were searched through the search engine. The search scope was set to “all fields” to minimize the risk of overlooking potential articles. The search query consisted of two parts. In the first row (#1), the queried keywords were “artificial intelligence” OR “neural network” OR “deep learning” OR “machine learning” OR “data mining” OR “big data” (All fields search). In the second row (#2), the queried keywords were “orthopaedics” OR “orthopaedic” OR “orthopedics” OR “orthopedic” OR “sports medicine” OR “fracture” OR “trauma” OR “osteoarthritis” OR “bone cancer” OR “arthroplasty” OR “joint replacement” (All fields search). The final dataset was #1 AND #2. Articles indexed in the Science Citation Index Expanded (SCI-Expanded) or Emerging Sources Citation Index (ESCI) (Web of Science Index) were included. No language restriction was applied. The search was conducted on August 17, 2023, within a single day, as a precaution against potential updates of the literature. No restrictions were applied regarding the publication date of the articles, resulting in a total of 5,561 records. The articles were ranked based on the number of citations from the most to the least (Figure 1). For sensitivity in the research, two independent researchers (NT, SD) evaluated the titles, abstracts and full texts of the articles to be included. The articles were screened to ensure their relevance to the field of orthopedics. Upon evaluation of the articles obtained from the search, if an article was deemed to be an artificial intelligence study unrelated to the field of orthopedics, it was excluded from the study. The top 100 most cited articles were identified and exported in plain text format for further analysis. Result analyses were performed in Web of Science. These articles were assessed in terms of authors, citations, publication year, country/region, area of orthopedics, institutions, journals, the annual number of published articles and funding agencies. The impact factor values of the journals were obtained from the 2022 Journal Citation Reports (JCR). The country of the first author was considered as the country of origin for each respective article. VOSviewer (version 1.6.19) was used for the advanced bibliometric analysis.

### Data analysis

VOSviewer is a bibliometric tool that facilitates quantitative analysis of the literature. This tool enables the generation of co-occurrence, co-citation, and co-authorship maps for the defined dataset. Keywords were assessed using co-occurrence analysis. Keyword co-occurrence analysis examines how many times a pair of keywords are mentioned within the same article. For citation analysis, references, cited authors, and journals were selected. If a third article references two other articles in its reference list, then these two referenced articles have a co-citation relationship with each other. Authors and institutions were evaluated through cooperative network analysis.

## Results

In this study, we compiled the top 100 most cited articles in the field of orthopedics related to artificial intelligence (AI). The articles were presented in descending order based on their citation number (Table 1). It was observed that the number of citations of these articles ranged from 32 to 272, with six papers having more than 200 citations. The article titled “Deep-learning-assisted diagnosis for knee magnetic resonance imaging: Development and retrospective validation of MRNet” by Bien et al. was the most cited and published in PLOS Medicine journal, with an annual citation rate of 55.4 (11). The average citation count for the articles in the Top 100 list was determined to be 72.35.

**Table 1:** The most cited 100 articles in orthopedics related to artificial intelligence.

Rank	Authors	Publication journal	Article Title	Times cited, WoS core	Publication years	Annual citation count
1	Bien, N	PLoS Medicine	Deep-learning-assisted diagnosis for knee magnetic resonance imaging: Development and retrospective validation of MRNet	277	2018	55.4
2	Lindsey, R	Proceedings of the National Academy of Sciences of The United States of America	Deep neural network improves fracture detection by clinicians	268	2018	53.6
3	Tiulpin, A	Scientific Reports	Automatic Knee Osteoarthritis Diagnosis from Plain Radiographs: A Deep Learning-Based Approach	248	2018	49.6
4	Olczak, J	Acta Orthopaedica	Artificial intelligence for analyzing orthopedic trauma radiographs Deep learning algorithms-are they on par with humans for diagnosing fractures?	217	2017	36.2
5	Kim, DH	Clinical Radiology	Artificial intelligence in fracture detection: transfer learning from deep convolutional neural networks	213	2018	42.6
6	Bini, SA	Journal of Arthroplasty	Artificial Intelligence, Machine Learning, Deep Learning, and Cognitive Computing: What Do These Terms Mean and How Will They Impact Health Care?	207	2018	41.4
7	Chung, SW	Acta orthopaedica	Automated detection and classification of the proximal humerus fracture by using deep learning algorithm	195	2018	39,0
8	Rutledge, R	Journal of Trauma-Injury Infection and Critical Care	The end of the Injury Severity Score (ISS) and the Trauma and Injury Severity Score (TRISS): ICISS, an International Classification of Diseases, Ninth Revision-Based Prediction Tool, outperforms both ISS and TRISS as predictors of trauma patient survival, hospital charges, and hospital length of stay	178	1998	7.1
9	Deluzio, KJ	Human Movement Science	Principal component models of knee kinematics and kinetics: Normal vs. pathological gait patterns	145	1997	5.7
10	Liu, F	Radiology	Deep Learning Approach for Evaluating Knee MR Images: Achieving High Diagnostic Performance for Cartilage Lesion Detection	136	2018	27.2
11	Helm, JM	Current Reviews in Musculoskeletal Medicine	Machine Learning and Artificial Intelligence: Definitions, Applications, and Future Directions	131	2020	43.7
12	Ben-Ari, A	Journal of Bone and Joint Surgery-American Volume	Preoperative Opioid Use Is Associated with Early Revision After Total Knee Arthroplasty A Study of Male Patients Treated in the Veterans Affairs System	125	2017	20.8
13	Cheng, CT	European Radiology	Application of a deep learning algorithm for detection and visualization of hip fractures on plain pelvic radiographs	120	2019	30
14	Karatsidis, A	Sensors	Estimation of Ground Reaction Forces and Moments During Gait Using Only Inertial Motion Capture	118	2017	19.7
15	Tomita, N	Computers in Biology and Medicine	Deep neural networks for automatic detection of osteoporotic vertebral fractures on CT scans	114	2018	22.8
16	Urakawa, T	Skeletal Radiology	Detecting intertrochanteric hip fractures with orthopedist-level accuracy using a deep convolutional neural network	113	2019	28.2
17	Badgeley, MA	Npj Digital Medicine	Deep learning predicts hip fracture using confounding patient and healthcare variables	109	2019	27.2
18	Banerjee, R	Materials Science & Engineering C-Biomimetic and Supramolecular Systems	A novel combinatorial approach to the development of beta titanium alloys for orthopaedic implants	102	2005	5.7
19	Tiulpin, A	Scientific Reports	Multimodal Machine Learning-based Knee Osteoarthritis Progression Prediction from Plain Radiographs and Clinical Data	98	2019	24.5
20	Fontana, MA	Clinical Orthopaedics and Related Research	Can Machine Learning Algorithms Predict Which Patients Will Achieve Minimally Clinically Important Differences From Total Joint Arthroplasty?	96	2019	24
21	Kim, JS; Merrill, RK	Spine	Examining the Ability of Artificial Neural Networks Machine Learning Models to Accurately Predict Complications Following Posterior Lumbar Spine Fusion	87	2018	17.4
22	Jamshidi, A	Nature Reviews Rheumatology	Machine-learning-based patient-specific prediction models for knee osteoarthritis	82	2019	20.5
23	Burns, JE	Radiology	Vertebral Body Compression Fractures and Bone Density: Automated Detection and Classification on CT Images	81	2017	13.5
24	Thian, YL	Radiology-Artificial Intelligence	Convolutional Neural Networks for Automated Fracture Detection and Localization on Wrist Radiographs	80	2019	20
25	Harris, AHS	Clinical Orthopaedics and Related Research	Can Machine Learning Methods Produce Accurate and Easy-to-use Prediction Models of 30-day Complications and Mortality After Knee or Hip Arthroplasty?	79	2019	19.8
26	Navarro, SM	Journal of Arthroplasty	Machine Learning and Primary Total Knee Arthroplasty: Patient Forecasting for a Patient-Specific Payment Model	79	2018	15.8
27	Pierson, E	Nature Medicine	An algorithmic approach to reducing unexplained pain disparities in underserved populations	78	2021	39
28	Xue, YP	Plos One	A preliminary examination of the diagnostic value of deep learning in hip osteoarthritis	77	2017	12.8
29	Gan, KF	Acta Orthopaedica	Artificial intelligence detection of distal radius fractures: a comparison between the convolutional neural network and professional assessments	75	2019	18.8
30	Brahim, A	Computerized Medical Imaging and Graphics	A decision support tool for early detection of knee OsteoArthritis using x-ray imaging and machine learning: Data from the OsteoArthritis Initiative	71	2019	17.8
31	Pedoia, V	Journal of Magnetic Resonance Imaging	3D convolutional neural networks for detection and severity staging of meniscus and PFJ cartilage morphological degenerative changes in osteoarthritis and anterior cruciate ligament subjects	71	2019	17.8
32	Ramkumar, PN	Journal of Arthroplasty	Development and Validation of a Machine Learning Algorithm After Primary Total Hip Arthroplasty: Applications to Length of Stay and Payment Models	70	2019	17.8
33	Norman, BS	Journal of Digital Imaging	Applying Densely Connected Convolutional Neural Networks for Staging Osteoarthritis Severity from Plain Radiographs	68	2019	17
34	Karhade, AV	Journal of Arthroplasty	Development of Machine Learning Algorithms for Prediction of Sustained Postoperative Opioid Prescriptions After Total Hip Arthroplasty	67	2019	16.8
35	Chen, PJ	Computerized Medical Imaging and Graphics	Fully automatic knee osteoarthritis severity grading using deep neural networks with a novel ordinal loss	67	2019	16.8
36	Galbusera, F	European Spine Journal	Fully automated radiological analysis of spinal disorders and deformities: a deep learning approach	66	2019	16.5
37	Ramkumar, PN	Journal of Arthroplasty	Remote Patient Monitoring Using Mobile Health for Total Knee Arthroplasty: Validation of a Wearable and Machine Learning-Based Surveillance Platform	64	2019	16
38	Leung, K	Radiology	Prediction of Total Knee Replacement and Diagnosis of Osteoarthritis by Using Deep Learning on Knee Radiographs: Data from the Osteoarthritis Initiative	63	2020	21
39	Liu, F	Radiology-Artificial Intelligence	Fully Automated Diagnosis of Anterior Cruciate Ligament Tears on Knee MR Images by Using Deep Learning	61	2019	15.3
40	Adams, M	Journal of Medical Imaging and Radiation Oncology	Computer vs. human: Deep learning versus perceptual training for the detection of neck of femur fractures	61	2019	15.3
41	Pranata, YD	Computer Methods And Programs In Biomedicine	Deep learning and SURF for automated classification and detection of calcaneus fractures in CT images	58	2019	14.5
42	Thio, QCBS	Clinical Orthopaedics and Related Research	Can Machine-learning Techniques Be Used for 5-year Survival Prediction of Patients With Chondrosarcoma?	58	2018	11.6
43	Loffler, MT	Radiology-Artificial Intelligence	A Vertebral Segmentation Dataset with Fracture Grading	56	2020	18.7
44	Kitamura, G	Journal of Digital Imaging	Ankle Fracture Detection Utilizing a Convolutional Neural Network Ensemble Implemented with a Small Sample, De Novo Training, and Multiview Incorporation	56	2019	14.5
45	Kotti, M	Medical Engineering & Physics	Detecting knee osteoarthritis and its discriminating parameters using random forests	56	2017	9.3
46	Muehlematter, UJ	European Radiology	Vertebral body insufficiency fractures: detection of vertebrae at risk on standard CT images using texture analysis and machine learning	55	2019	13.8
47	Merali, ZG	PLoS One	Using a machine learning approach to predict outcome after surgery for degenerative cervical myelopathy	54	2019	10.8
48	Ratliff, JK	Journal of Bone and Joint Surgery-American Volume	Predicting Occurrence of Spine Surgery Complications Using Big Data Modeling of an Administrative Claims Database	53	2016	7.6
49	Karhade, AV	Spine Journal	Development of machine learning algorithms for prediction of prolonged opioid prescription after surgery for lumbar disc herniation	51	2019	12.8
50	Mutasa, S	Journal of Digital Imaging	MABAL: a Novel Deep-Learning Architecture for Machine-Assisted Bone Age Labeling	51	2018	10.2
51	Ashinsky, BG	Journal of Orthopaedic Research	Predicting Early Symptomatic Osteoarthritis in the Human Knee Using Machine Learning Classification of Magnetic Resonance Images From the Osteoarthritis Initiative	51	2017	8.5
52	RUTLEDGE, R	Journal of Trauma-Injury Infection and Critical Care	Injury Severity and Probability of Survival Assessment in Trauma Patients Using a Predictive Hierarchical Network Model Derived From ICD-9 Codes	51	1995	1.8
53	Krogue, JD	Radiology-Artificial Intelligence	Automatic Hip Fracture Identification and Functional Subclassification with Deep Learning	50	2020	16.7
54	Raghavendra, U	Future Generation Computer Systems-the International Journal of Escience	Automated system for the detection of thoracolumbar fractures using a CNN architecture	50	2018	10
55	Kruse, C	Calcified Tissue International	Machine Learning Principles Can Improve Hip Fracture Prediction	50	2017	8.3
56	Tolpadi, AA	Scientific Reports	Deep Learning Predicts Total Knee Replacement from Magnetic Resonance Images	49	2020	16.3
57	Jones, GG	Bone & Joint Journal	Gait comparison of unicompartmental and total knee arthroplasties with healthy controls	49	2016	7.6
58	Keijsers, NLW	Clinical Biomechanics	Classification of forefoot pain based on plantar pressure measurements	49	2013	4.9
59	Bissonnette, V	Journal of Bone and Joint Surgery-American Volume	Artificial Intelligence Distinguishes Surgical Training Levels in a Virtual Reality Spinal Task	48	2019	12
60	Huber, M	Bmc medical Informatics and Decision Making	Predicting patient-reported outcomes following hip and knee replacement surgery using supervised machine learning	48	2019	12
61	Abidin, AZ	Computers in Biology and Medicine	Deep transfer learning for characterizing chondrocyte patterns in phase contrast x-Ray computed tomography images of the human patellar cartilage	48	2018	9.6
62	Auloge, P	European Spine Journal	Augmented reality and artificial intelligence-based navigation during percutaneous vertebroplasty: a pilot randomised clinical trial	47	2020	15.7
63	Jones, RM	NPJ Digital Medicine	Assessment of a deep-learning system for fracture detection in musculoskeletal radiographs	46	2020	15.3
64	Ramkumar, PN	Journal of Arthroplasty	Deep Learning Preoperatively Predicts Value Metrics for Primary Total Knee Arthroplasty: Development and Validation of an Artificial Neural Network Model	46	2019	11.5
65	Bedard, NA	Journal of Arthroplasty	Big Data and Total Hip Arthroplasty: How Do Large Databases Compare?	46	2018	9.2
66	Yamamoto, N	Biomolecules	Deep Learning for Osteoporosis Classification Using Hip Radiographs and Patient Clinical Covariates	45	2020	15
67	Pedoia, V	Osteoarthritis and Cartilage	Diagnosing osteoarthritis from T-2 maps using deep learning: an analysis of the entire Osteoarthritis Initiative baseline cohort	44	2019	11
68	Ferizi, U	Journal of Magnetic Resonance Imaging	Artificial Intelligence Applied to Osteoporosis: A Performance Comparison of Machine Learning Algorithms in Predicting Fragility Fractures From MRI Data	44	2019	11
69	Rayan, JC	Radiology-Artificial Intelligence	Binomial Classification of Pediatric Elbow Fractures Using a Deep Learning Multiview Approach Emulating Radiologist Decision Making	44	2019	11
70	Borjali, A	Journal of Orthopaedic Research	Detecting total hip replacement prosthesis design on plain radiographs using deep convolutional neural network	42	2020	14
71	Du, YD	IEEE Transactions on Nanobioscience	A Novel Method to Predict Knee Osteoarthritis Progression on MRI Using Machine Learning Methods	42	2018	8.4
72	Yoo, TK	Yonsei Medical Journal	Osteoporosis Risk Prediction for Bone Mineral Density Assessment of Postmenopausal Women Using Machine Learning	42	2013	4.2
73	Lin, CC	Injury-International Journal of the Care of the Injured	Comparison of artificial neural network and logistic regression models for predicting mortality in elderly patients with hip fracture	42	2010	3.2
74	Shohat, N	Bone & Joint Journal	2020 Frank Stinchfield Award: Identifying who will fail following irrigation and debridement for prosthetic joint infection A MACHINE LEARNING-BASED VALIDATED TOOL	40	2020	13.3
75	Thomas, KA	Radiology-Artificial Intelligence	Automated Classification of Radiographic Knee Osteoarthritis Severity Using Deep Neural Networks	40	2020	13.3
76	Choi, JW	Investigative Radiology	Using a Dual-Input Convolutional Neural Network for Automated Detection of Pediatric Supracondylar Fracture on Conventional Radiography	40	2020	13.3
77	Stajduhar, I	Computer Methods and Programs in Biomedicine	Semi-automated detection of anterior cruciate ligament injury from MRI	40	2017	6.7
78	Tiulpin, A	Diagnostics	Automatic Grading of Individual Knee Osteoarthritis Features in Plain Radiographs Using Deep Convolutional Neural Networks	39	2020	13
79	Yi, PH	Knee	Automated detection & classification of knee arthroplasty using deep learning	39	2020	13
80	Lim, J	International Journal of Environmental Research and Public Health	A Deep Neural Network-Based Method for Early Detection of Osteoarthritis Using Statistical Data	38	2019	9.5
81	Azimi, P	Journal of Neurosurgery-Spine	Use of artificial neural networks to predict surgical satisfaction in patients with lumbar spinal canal stenosis	38	2014	4.2
82	Ottenbacher, KJ	Annals of Epidemiology	Comparison of logistic regression and neural network analysis applied to predicting living setting after hip fracture	38	2004	2
83	Schmaranzer, F	Clinical Orthopaedics and Related Research	Automatic MRI-based Three-dimensional Models of Hip Cartilage Provide Improved Morphologic and Biochemical Analysis	37	2019	9.3
84	Guermazi, A	Radiology	Improving Radiographic Fracture Recognition Performance and Efficiency Using Artificial Intelligence	36	2022	36
85	Bowes, MA	Annals of the Rheumatic Diseases	Machine-learning, MRI bone shape and important clinical outcomes in osteoarthritis: data from the Osteoarthritis Initiative	36	2021	18
86	Bini, SA	Journal of Arthroplasty	Machine Learning Algorithms Can Use Wearable Sensor Data to Accurately Predict Six-Week Patient-Reported Outcome Scores Following Joint Replacement in a Prospective Trial	35	2019	8.8
87	Tanzi, L	European Journal of Radiology	Hierarchical fracture classification of proximal femur x-Ray images using a multistage Deep Learning approach	34	2020	11.3
88	Jo, C	Knee Surgery Sports Traumatology Arthroscopy	Transfusion after total knee arthroplasty can be predicted using the machine learning algorithm	34	2020	11.3
89	von Schacky, CE	Radiology	Development and Validation of a Multitask Deep Learning Model for Severity Grading of Hip Osteoarthritis Features on Radiographs	34	2020	11.3
90	Nwachukwu, BU	American Journal of Sports Medicine	Application of Machine Learning for Predicting Clinically Meaningful Outcome After Arthroscopic Femoroacetabular Impingement Surgery	34	2020	11.5
91	Gowd, AK	Journal of Shoulder and Elbow Surgery	Construct validation of machine learning in the prediction of short-term postoperative complications following total shoulder arthroplasty	34	2019	8.5
92	Pedoia, V	Journal of Magnetic Resonance Imaging	MRI and biomechanics multidimensional data analysis reveals R-2-R-1 as an early predictor of cartilage lesion progression in knee osteoarthritis	34	2018	6.8
93	Widera, P	Scientific Reports	Multi-classifier prediction of knee osteoarthritis progression from incomplete imbalanced longitudinal data	33	2020	11
94	Kunze, KN	Journal of Arthroplasty	Development of Machine Learning Algorithms to Predict Patient Dissatisfaction After Primary Total Knee Arthroplasty	32	2020	10.7
95	Lee, S	Skeletal Radiology	The exploration of feature extraction and machine learning for predicting bone density from simple spine x-ray images in a Korean population	32	2020	10.7
96	Ramkumar, PN	Journal of Arthroplasty	Preoperative Prediction of Value Metrics and a Patient-Specific Payment Model for Primary Total Hip Arthroplasty: Development and Validation of a Deep Learning Model	32	2019	8
97	Cilla, M	Plos One	Machine learning techniques for the optimization of joint replacements: Application to a short-stem hip implant	32	2017	5.3
98	Yu, XH	Neurocomputing	Application of artificial neural network in the diagnostic system of osteoporosis	32	2016	4.6
99	Yoo, TK	Plos One	Simple Scoring System and Artificial Neural Network for Knee Osteoarthritis Risk Prediction: A Cross-Sectional Study	32	2016	4.6
100	Atkinson, EJ	Journal of Bone and Mineral Research	Assessing fracture risk using gradient boosting machine (GBM) models	32	2012	2.9

WoS, Web of Science.

**Figure 2:**
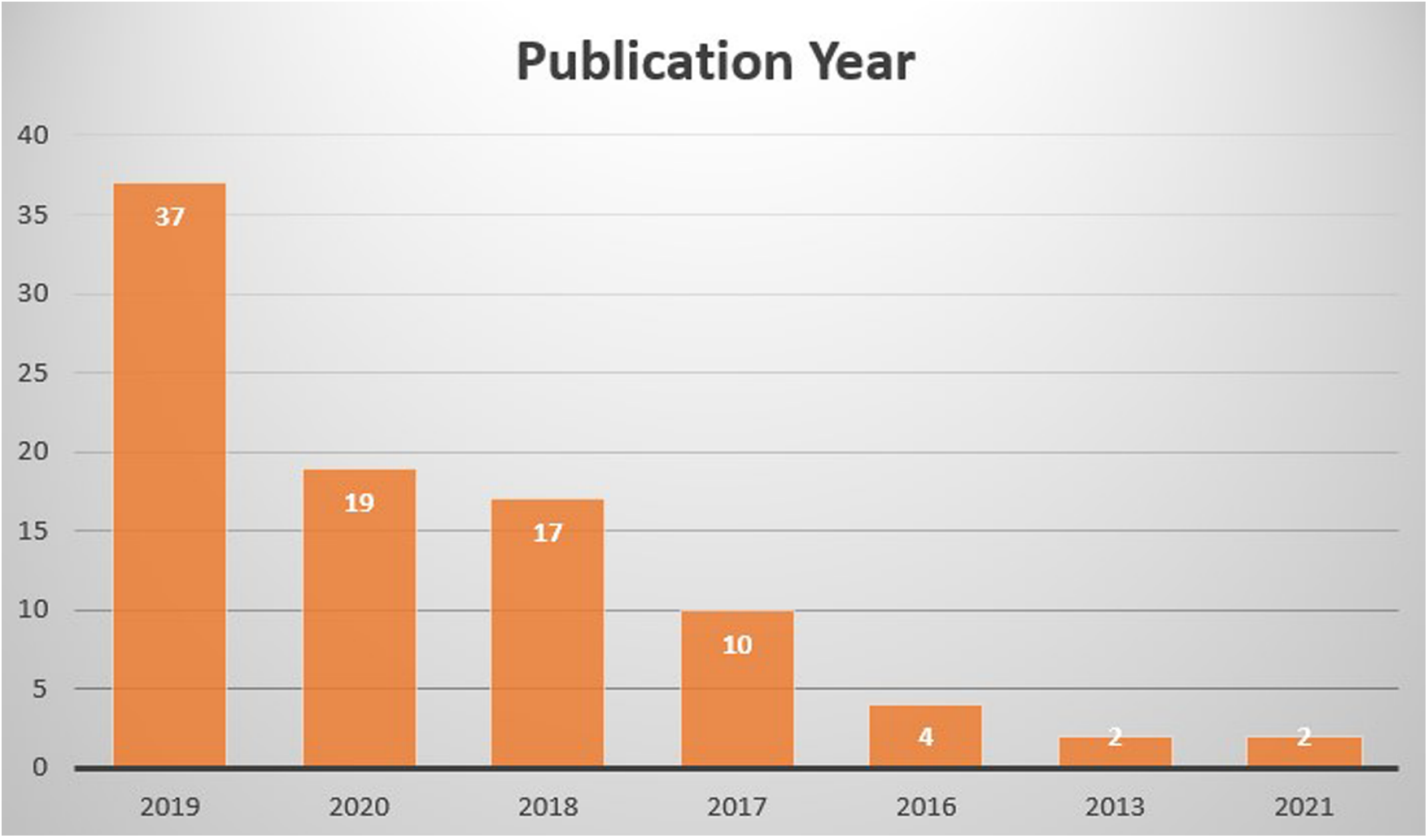
Publication years of articles.

The articles within the Top 100 list were published between 1995 and 2022, spanning over a 27-year period (Figure 2). When examined by decades, there were 3 articles from the 1990s, 2 from the 2000s, 73 from the 2010s, and 22 from the 2020s. The most highly cited articles were predominantly published in 2019 (*n*: 37) and 2020 (*n*: 19), comprising 56% of the Top 100 list. Even though they have been recently published, two articles from 2021 and one from 2022 were able to be included in this list. The oldest paper was published in 1995 with a title of “Injury severity and probability of survival assessment in trauma patients using a predictive hierarchical network model derived from ICD-9 codes” in Journal of Trauma-Injury Infection and Critical Care (12). Robert Rutledge used neural networks for computer-assisted decision-making in this study.

The articles originated from 28 different countries, with the USA (*n*: 61) contributing significantly to the literature in terms of publication volume (Table 2). England (*n*: 12) was the second, followed by others including Germany (*n*: 7), Netherlands (*n*: 6), Canada (*n*: 5), and France (*n*: 5).

**Table 2:** Ranking of the countries based on the number of publications (the countries with ≥ 3 publications.

Rank	Countries/regions	Number of publications
1	USA	61
2	England	12
3	Germany	7
3	South Korea	7
5	Netherlands	6
6	Canada	5
6	France	5
8	Finland	4
8	Peoples R China	4
10	Taiwan	3

These articles were published in 55 different journals. “The Journal of Arthroplasty”, which had an impact factor of 3.5 in 2022, was the most published journal title with ten articles, while “Radiology Artificial Intelligence” had six articles, “Radiology” had five, and “Clinical Orthopaedics And Related Research,” “PLOS ONE,” and “Scientific Reports” had four each. The journals with two or more articles and their respective impact factors are presented in Table 3. The journal with the biggest impact factor was “Radiology”.

**Table 3:** The most published journals of top 100 list (with ≥2 publications).

Publication Titles	Record count	IF score
Journal of Arthroplasty	10	3.5 (2.8)
Radiology Artificial Intelligence	6	9.8 (9.7)
Radiology	5	19.7 (19.1)
Clinical Orthopaedics and Related Research	4	4.2 (3.7)
Plos One	4	3.7 (3.5)
Scientific Reports	4	4.6 (4.4)
Acta Orthopaedica	3	3.7 (3.4)
Journal of Bone and Joint Surgery American Volume	3	5.3 (4.9)
Journal of Digital Imaging	3	4.4 (4.1)
Journal of Magnetic Resonance Imaging	3	4.4 (4.1)
Bone Joint Journal	2	4.6 (4.1)
Computer Methods and Programs in Biomedicine	2	6.1 (5.7)
Computerized Medical Imaging and Graphics	2	5.7 (5.5)
Computers in Biology and Medicine	2	7.7 (6.9)
European Radiology	2	5.9 (5.4)
European Spine Journal	2	2.8 (2.5)
Journal of Orthopaedic Research	2	2.8 (2.7)
Journal of Trauma Injury Infection and Critical Care	2	3.0*
Npj Digital Medicine	2	15.2 (14.7)
Skeletal Radiology	2	2.1(1.9)

IF, impact factor.

* The recent impact factor was in 2013.

() It marks the impact factor without self-citations.

A total of 608 authors contributed to the Top 100 list. Among the high-yield authors with the most cited articles, four individuals led the list with contributions to six articles each: Haeberle HS, Majumdar S, Pedoia V, and Ramkumar PN (Table 4). All four authors were affiliated with institutions in the USA. Both Haeberle Hs and Ramkumar PN have received a total of 422 citations. A total of 250 different institutions contributed to the Top 100 list. The institutions with highest number of publications were University of California System (*n*: 13), followed by University of California San Francisco (*n*: 10), Harvard University (*n*: 9), Stanford University (*n*: 9), Baylor College of Medicine (*n*: 8), and Cleveland Clinic Foundation (*n*: 7).

**Table 4:** Ranking of the authors based on the number of publications (the authors with ≥5 publications).

Authors	Number of publication
Haeberle HS	6
Majumdar S	6
Pedoia V	6
Ramkumar PN	6
Krebs VE	5
Mont MA	5
Navarro SM	5
Patterson BM	5

The majority of articles, 96%, had three or more authors. Two articles were single-authored (Bini, Stefano A.; Rutledge, R), and two were co-authored by two individuals. Funding agencies were acknowledged in 55 articles. The most prominent contributors included the United States Department Of Health Human Services (*n*: 18), National Institutes Of Health NIH USA (*n*: 17), GlaxoSmithKline (*n*: 7), Merck Company (*n*: 7), Novartis (*n*: 7), and Pfizer (*n*: 7). Sixty articles were categorized as “all open access.”

Among the articles, 94 were found in SCI-expanded journals (*n*: 94), while 6 were in ESCI journals. Of the total, 97 articles were categorized as original studies, with only 3 articles identified as reviews. Only five of the studies were classified as clinical studies which involved people. The ninety-seven original articles were categorized according to the themes of the researches (Table 5). Decision support systems were used in a quarter of the list (*n*: 25). Fracture detection (*n*: 24) was the second most researched subject. They were followed by osteoarthritis and its staging (*n*: 21). The other themes were intraarticular joint evaluation (*n*: 8), osteoporosis (*n*: 6), gait analysis (*n*: 3), biomaterials (2), implant identification (*n*: 2), determination of bone age (*n*: 1), surgery which was vertebroplasty (*n*: 1), detection of spinal disorders and deformities (*n*: 1), trauma scoring systems (*n*: 1), education (*n*: 1), remote monitoring after arthroplasty (*n*: 1).

**Table 5:** The classification of the themes of the articles.

Rank	Theme	Number of articles
1	Decision support systems (prediction of patient satisfaction, patient payment, complications)	25
2	Fracture detection	24
3	Osteoarthritis detection, grading severity, prediction of progression	21
4	Diagnosing of intraarticular knee and hip pathologies	8
5	Osteoporosis	6
6	Gait analysis	3
7	Biomaterials	2
8	Implant identification	2
9	Education	1
10	Bone age determination	1
11	Identification of spinal disorders and deformities	1
12	Trauma scoring systems	1
13	Surgery (percutaneous vertebroplasty)	1
14	Remote monitoring after arthroplasty	1

The studies were predominantly conducted for diagnostic purposes (*n*: 85), with only one study conducted for therapeutic purposes (Table 6). Fracture diagnosis studies involved the use of CT in only two articles, with the remaining 23 articles relying on x-ray images. All eight articles, which investigate intraarticular structure pathologies, used MRI scanning. In four of the osteoarthritis diagnosis and staging studies, MRI imaging was used, while x-ray images were employed in the remaining 17 research studies.

**Table 6:** The classification of the objectives of the articles.

Rank	Objective of the study	Number of articles
1	Diagnosis	85
2	Basic sciences	5
3	Gait analysis	3
4	Therapeutic	2
5	Trauma scoring	2
6	Treatment follow-up	2
7	Education	1

Regarding the Top 100 list, it was observed that only 38 articles were published in the WOS orthopedics category, while the majority were published in journals from other fields. The most closely related fields on this topic were radiology, nuclear medicine, surgery, and computer science.

### Bibliometric co-occurrence analysis

**Figure 3:**
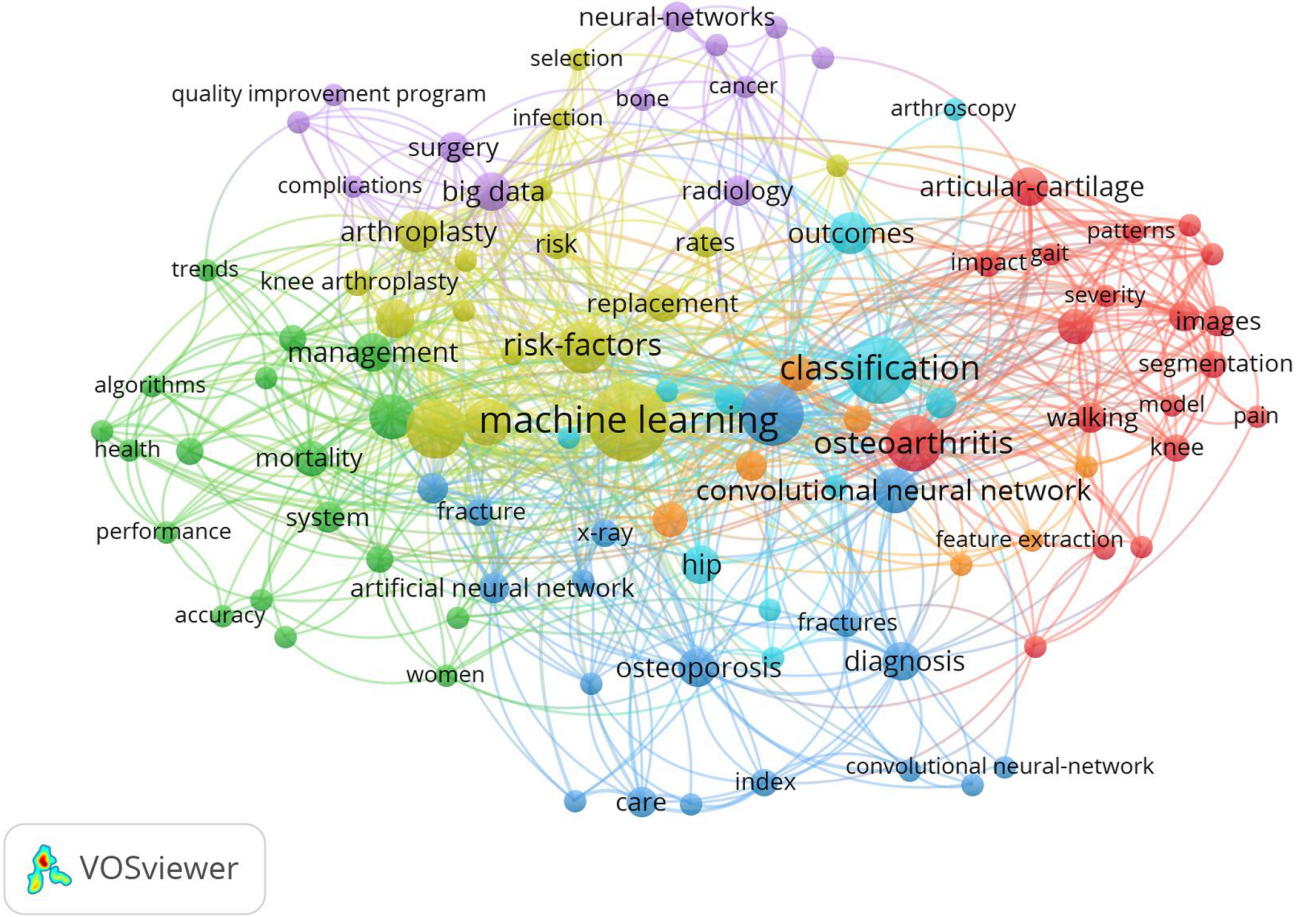
Mapping on co-occurrence of keywords related to orthopedics and traumatology with artificial intelligence. Each point represented in different colors and sizes represents a keyword. An increase in the size of a point signifies a higher frequency of usage for that keyword. The line connecting two points indicates that both keywords appeared in the same article. The figure was created using VOSviewer (version 1.6.19) software.

An analysis was conducted on the 497 keywords obtained from the studies in this list. The minimum number of occurrences of a keyword was set at two. 102 keywords met the criteria. The keywords were analyzed and visualized by VOSviewer software (Figure 3). Each keyword is represented by a circle in the figure. The keywords that co-occur more frequently are displayed with larger circles. The most frequently used keywords were “machine learning” (*n*: 26), “classification” (*n*: 18), “deep learning” (*n*: 16), “artificial intelligence” (*n*: 14), respectively. The other keywords with the highest occurrences comprising the top ten were “osteoarthritis”, “risk factors”, “prediction”, “arthroplasty”, “artificial-intelligence”, and “convolutional neural network”, respectively (Table 7).

**Table 7:** Top ten keywords.

Rank	Keyword	Count	Total link strength
1	Machine learning	26	134
2	Classification	18	73
3	Deep learning	16	67
4	Artificial intelligence	14	69
5	Osteoarthritis	13	60
6	Risk-factors	11	52
7	Prediction	9	47
8	Artificial-intelligence	8	32
9	Convolutional neural network	8	32
10	Arthroplasty	7	27

### Bibliometric co-citation analysis

**Figure 4:**
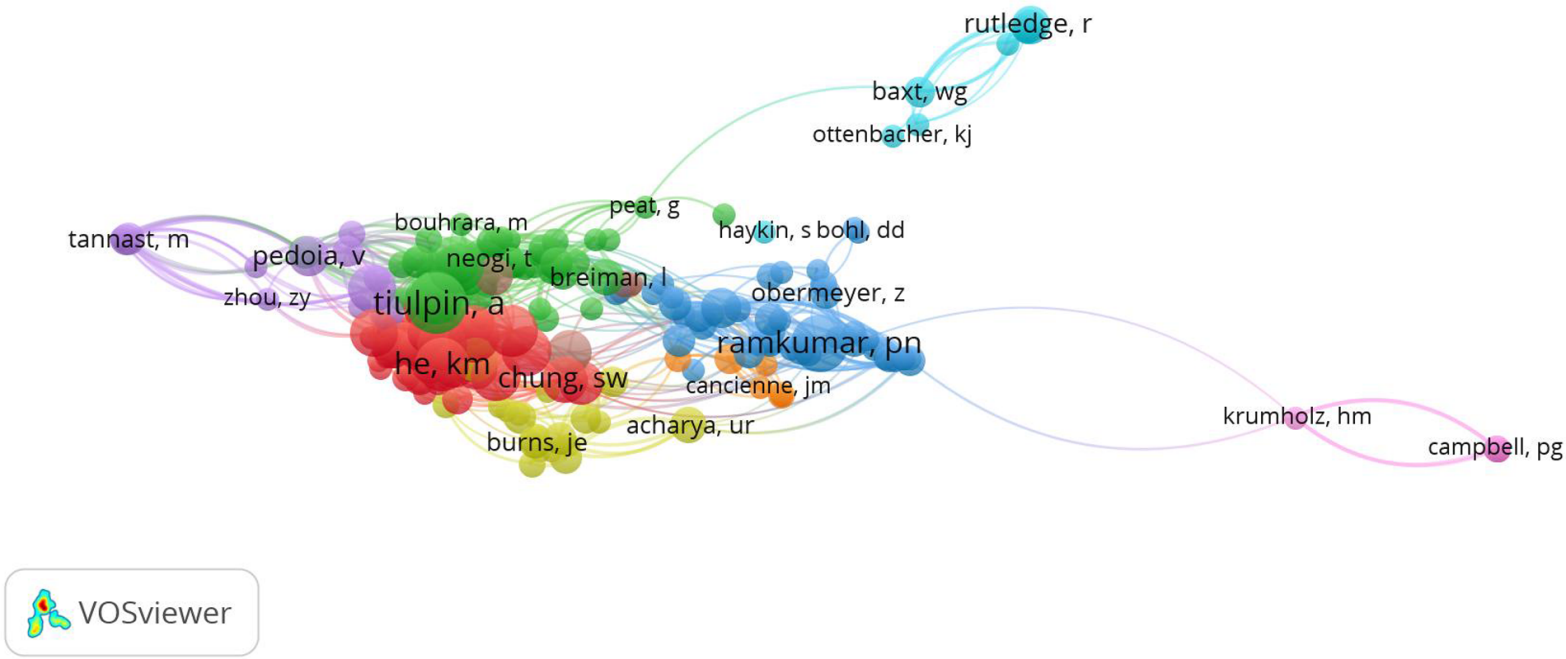
Mapping on co-cited authors of publications related to orthopedics and traumatology with artificial intelligence. The varying colors represent different authors who were cited. An increase in the size of a point signifies a higher citation frequency of the author. The line connecting two points indicates that both authors had been cited in the same article. The length of a line indicates the proximity between two authors; the closer the link, the shorter the line. The figure was created using VOSviewer (version 1.6.19) software.

Co-citations of authors were analyzed. The mapping is shown in Figure 4. When the link between two authors is stronger, they appear closer to each other, and the line between them is thicker. If there is a line between two authors, it signifies that they are referenced in the same article. The minimum number of citations of an author was set at three, and 222 authors met the threshold of the 2,309 authors. Tiulpin, A had the most citations of 20 and total link strength of 350.

**Figure 5:**
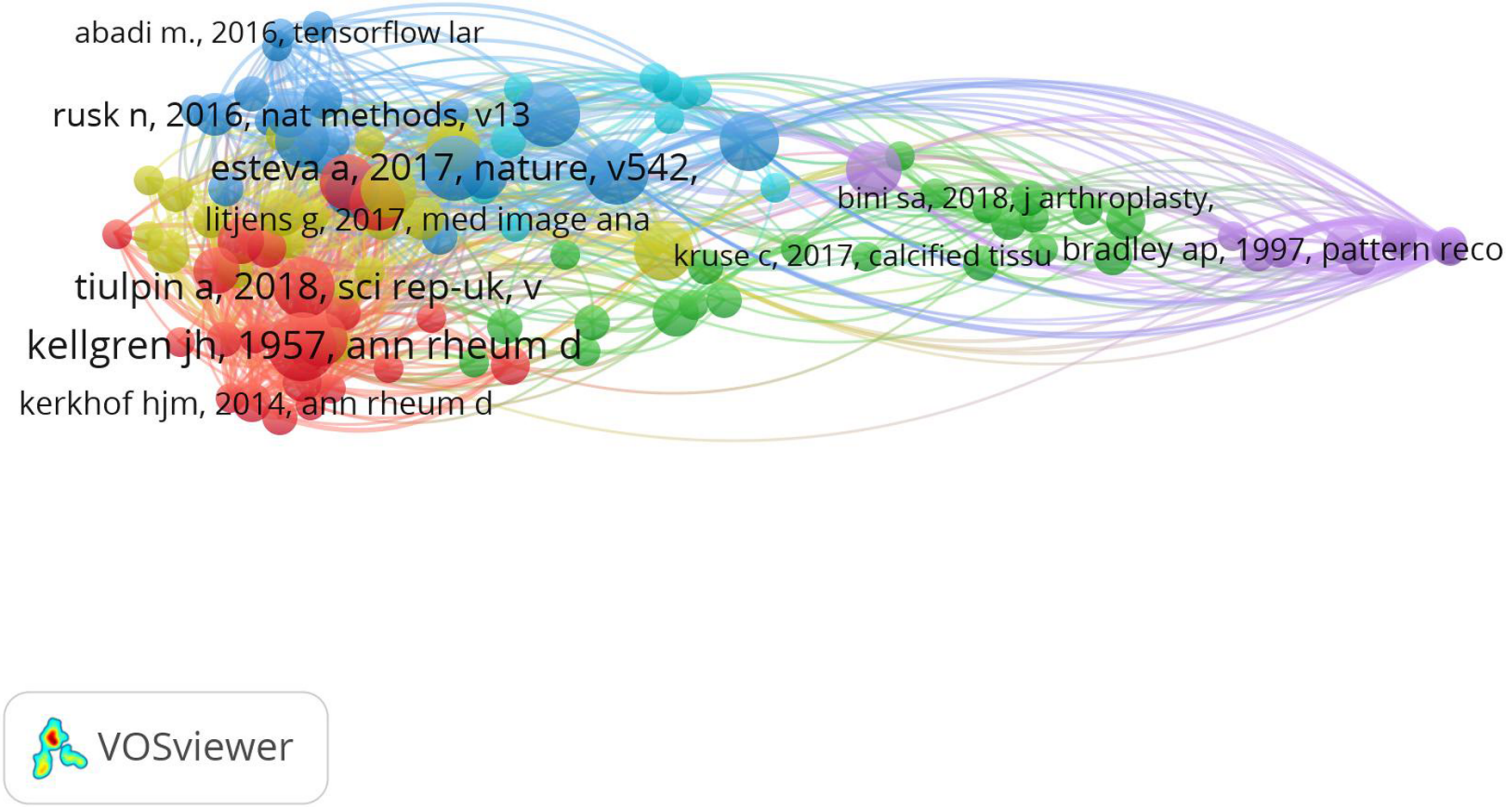
Mapping on co-cited references of publications related to orthopedics and traumatology with artificial intelligence. The varying colors represent different articles which were cited and categorized the articles into distinct clusters. An increase in the size of a point signifies a higher citation frequency of the articles. The line connecting two points indicates that both references had been cited in the same article. The length of a line indicates the proximity between two articles; the closer the link, the shorter the line. The figure was created using VOSviewer (version 1.6.19) software.

Co-citations of references are beneficial for assessing the presence of similarity by determining the number of articles in which these references are collectively cited. The minimum number of citations of a cited article was set at three, and 112 met the threshold of the 2,874 cited references. The article “Radiological assessment of osteo-arthrosis” by JH Kellgren and JS Lawrence had the most citations of 15 (13). The visual analysis is shown in Figure 5.

## Discussion

This bibliometric analysis provides valuable insights into the literature related to the utilization of artificial intelligence in orthopedics up until the present day. The authors of the most cited articles, journal names, countries, and other key characteristics were highlighted. The results of this article indicate that research on artificial intelligence applications in orthopedics will continue to accelerate. The rapid increase in the number of publications in recent years, along with the fact that the most cited articles are closely aligned with the year 2019, serves as indicators of this trend. Interestingly, when considering only the years 2019 and 2020 together, we observed that 56 articles from the list were published during these two years. This observation confirms our initial hypothesis. As far as we know, this is the first study to examine the top 100 most cited articles related to artificial intelligence in orthopedic research. The findings from this article will provide valuable insights for future studies and guide researchers interested in this subject.

When examining the countries where the research was conducted, it is evident that developed countries, especially the USA, have significantly contributed to the literature. We observed that most of the top 100 articles (61 of them) originated solely from the USA. Countries’ support and funding for artificial intelligence research play a crucial role in this distribution. In the realm of artificial intelligence applications in other medical fields as well, the USA leads in academic output (14, 15). The nearest contributor, the England, has contributed less than 20% of the USA's publications. The level of support for science is directly proportional to a country's level of development. As global prosperity increases, other countries will also contribute more to publications related to the use of artificial intelligence in orthopedics, accelerating the integration of artificial intelligence applications into clinical practice.

Keyword analysis reveals that the prominent keywords are “machine learning,” “deep learning,” “artificial intelligence,” and “classification.” Co-occurrence keyword analysis indicates that “machine learning” is the most frequently used keyword. Machine learning, a subset of artificial intelligence, revolves around the principle of enabling virtual machines to learn from data without explicit programming. Machine learning has advanced the field by allowing preoperative risk analysis, outcome prediction, and mortality rate determination.

Fracture recognition systems can lead to a dramatic reduction in misdiagnosis rates and treatment deficiencies. Fracture recognition systems are among the most studied areas of machine learning, focusing particularly on hip and wrist fractures (16, 17). The increasing incidence of osteoporosis due to an aging population has led to a rise in these fracture types, which are the most common and have significant morbidity and mortality. Fracture detection using artificial neural networks minimizes human error. Similarly, arthrosis staging systems can lead to more accurate treatments in orthopedic and rheumatology fields through early diagnosis and precise staging. One of the fundamental issues in classification systems is the challenges encountered in interobserver and intraobserver reliability tests (18). The primary goal of classification systems is to guide treatment. Thus, even though these studies appear to be focused on classification, their true objective is to pave the way for more successful treatments by developing control mechanisms for high-cost surgeries. While the current artificial intelligence studies in the field are primarily diagnostic and preoperative aids for surgeons, we anticipate that they will evolve to be more treatment-oriented and have a greater impact on intraoperative surgical management in the future. Risk classification systems will enable more personalized discussions with patients and their families based on clearer information, shedding light on potential complications and outcomes.

Direct radiographs have been in the foreground and predominantly used in both fracture recognition system and osteoarthritis classification system studies. The fact that direct radiography is the focus of studies can be explained by the fact that it is easier to work with a single two-dimensional cross-sectional image than MRI or CT.

When categorizing the listed studies for diagnostic and therapeutic purposes, we observed that most focused on diagnostic applications, particularly involving decision support systems. These studies predominantly pertain to radiology, which is consistent with the existing literature. In a conducted bibliometric analysis, the top 100 most cited articles in the healthcare field were compiled, and approximately one-fifth of these articles were found to be related to radiology (19). This can be attributed to the fact that radiology benefits from a multitude of artificial intelligence techniques. While fracture diagnosis constitutes the most common application within the field of orthopedics, other frequently employed aspects involve identifying intra-articular structures through MRI and evaluating structures through MRI, as well as the evaluation of structures such as the anterior cruciate ligament, meniscus, and cartilage. By using AI methods, AI models can be trained on large datasets, making pattern recognition and learning easier.

The boundaries of artificial intelligence extend beyond the scope of this study. Clinical intelligence and patient management, which clinicians develop over years in diagnosis and treatment, encompass human aspects that artificial intelligence cannot replicate. While the use of artificial intelligence, especially in robotic surgeries, is currently prominent in orthopedic practice, its impact is bound to increase significantly in everyday practice with technological advancement.

The rate of increase in articles related to artificial intelligence does not progress in direct proportion to its practical implications. In order for orthopedists and other medical professionals in various fields to integrate artificial intelligence applications into their daily practices, reliable clinical studies with high-level evidence, including trustworthy randomized controlled trials and meta-analyses, are required. To foster the adoption of artificial intelligence applications, substantial collaboration between computer science and medical experts is necessary, which in turn elongates the process. With an augmentation in such collaboration, faster progress can be achieved.

There are certain limitations to this article. Firstly, while Web of Science is one of the top-quality search engines available, it does not encompass every journal in the literature, leading to the exclusion of some articles. The search strategy was designed considering the most frequently used potential keywords related to artificial intelligence, but articles using keywords not included in the search strategy may have been excluded. Another limitation is related to the determination of journals’ impact factors. The latest published 2022 Journal Citation Report has been used to determine journals' impact factors; however, it should be taken into account that this list is updated annually.

## Conclusion

This bibliometric study reveals that orthopedic research related to artificial intelligence predominantly focuses on fracture diagnosis and osteoarthritis classification. It can be predicted that research related to bone fractures and joint degeneration in different body regions will rise in the near future considering the rate of increase in the number of studies. The fact that these studies are funded by the countries producing the technology can be accepted as an indicator of the reflection on the number of studies. Currently, studies are predominantly focused on diagnosis and staging; however, over time, it is expected that the focus will shift towards treatment.

## Data Availability

The original contributions presented in the study are included in the article/Supplementary Material, further inquiries can be directed to the corresponding author.
